# Comparison of epidural, spinal, and saddle block for holmium laser enucleation of prostate (HoLEP): A prospective randomized, comparative study

**DOI:** 10.1097/MD.0000000000027534

**Published:** 2021-10-22

**Authors:** Young Hoon Kim, So Young Kwon, Eun Hwa Jun, Seung Tae Choi, Seong Jin Park, Yumi Kim

**Affiliations:** a Department of Anesthesiology and pain medicine, Seoul St. Mary's Hospital, College of Medicine, the Catholic University of Korea, Republic of Korea; b Department of Anesthesiology and pain medicine, St. Vincent's Hospital, College of Medicine, the Catholic University of Korea, Republic of Korea.

**Keywords:** benign prostatic hyperplasia, epidural block, holmium laser enucleation of prostate, saddle block, spinal block

## Abstract

**Background:**

Holmium laser enucleation of the prostate (HoLEP) has become an important treatment modality for benign prostate hypertrophy. The aim of the present study was to compare regional anesthesia methods for HoLEP operation and to determine the optimal technique.

**Methods:**

Sixty patients with American Society of Anesthesiologists scores of I-III were randomly allocated into 3 groups. Patients in group E received an epidural block with 75 mg of bupivacaine plus 50 μg of fentanyl. In group S, 15 mg of bupivacaine and 50 μg fentanyl were used for spinal anesthesia. In group SA, patients received saddle block with 15 mg of bupivacaine and 50 μg of fentanyl.

**Results:**

Time to T10 dermatome block and to maximal level block were longest in group E (*P* < .05), and maximal sensorial block level was higher in group E than group SA (*P* < .05). There was a significant difference in postoperative motor block, but no difference in systolic blood pressure and heart rate.

**Conclusion:**

Among 3 techniques, saddle block might be preferable in HoLEP because an adequate sensorial level was achieved with lower motor block and stable hemodynamics.

## 1 Introduction

Benign prostatic hyperplasia (BPH) is a common urologic disease in males, with up to 50% prevalence in men over the age of 50.^[1]^ BPH can cause physical obstruction of the prostatic urethra and result in lower urinary tract symptoms, such as irritation and obstruction.^[2]^ Transurethral resection of the prostate (TURP) is the generally accepted treatment of choice for BPH.

Holmium laser enucleation of the prostate (HoLEP) is a relatively modern technique,^[3]^ and the accumulated data have shown better results in long-term outcome, durability, safety and effectiveness of HoLEP compared with TURP and open prostatectomy.^[4]^ In terms of anesthetics, HoLEP surgery has several advantages, such as lower morbidity due to decreased transfusion rate and eliminating the risk of dilutional hyponatremia; however the disadvantages are associated with extended procedure time and steep learning curve.^[5,6]^

In TURP surgery, circulatory volume overload is possible due to excessive absorption of irrigation solution during the procedure, which can cause detrimental results in elderly patients who have a cardiopulmonary disorder. The prevalence of cardiac and pulmonary problems has relatively increased, and the mortality rate is 0.2% in patients who underwent TURP.^[7]^ Therefore, maintaining a stable hemodynamic status during anesthesia is important. Regional anesthesia is preferable because recognizing symptoms of TURP syndrome under general anesthesia is difficult. Furthermore, regional anesthesia helps post-operative analgesia, and reduces the need for tracheal intubation.

In several studies, types of regional anesthesia for TURP surgery have been compared.^[8,9]^ However, the anesthetic aspects of HoLEP have not been fully established, therefore, different types of anesthesia should be considered to determine the ideal anesthetic technique. In most published studies, the focus was on urological aspects of HoLEP.

The aim of the present study was to compare regional anesthesia methods for HoLEP operation and to determine the optimal regional anesthesia technique.

## 2 Materials and methods

### 2.1 Participants

A prospective, randomized, comparative study was performed after approval from the Institutional Review Board of Saint Vincent's Hospital, The Catholic University of Korea (VC14EISI0054). Sixty patients with American Society of Anesthesiologists Physical Status score I – III, scheduled for HoLEP to treat BPH in the Department of Urology, Saint Vincent's Hospital, were enrolled in the study. Patients, who had contraindications for regional anesthesia such as severe systemic infection or local infection at injection site, coagulopathy, or serious central nervous system of peripheral nerve disorders, and history of allergy to local anesthetics were excluded from the study, and also patients who rejected to regional anesthetic technique were not included. Patients taking anticoagulants stopped antithrombic agents before regional anesthesia according to recommendations of 2010 American Society of Regional Anesthesia and Pain Medicine guidelines.^[10]^ All include patients were fully active, and able to carry on all pre-disease performance without restriction.

### 2.2 Procedure

Upon arrival to the operating room, noninvasive blood pressure, heart rate, respiratory rate, and electrocardiogram were monitored. Using a sealed envelope method, patients were randomly divided into 3 groups (n = 20): epidural block (group E), spinal block (group S), and saddle block (group SA).

In group E, after the patient was placed in lateral decubitus position, skin was prepared with chlorohexidine and punctured using a 16 G Tuohy needle at L3 – 4 level. Loss of resistance technique was used to identify the epidural space. Next, 3 mL hyperbaric 0.5% bupivacaine (15 mg) was administered as a test dosage. After the epidural space was confirmed, 12 mL 0.5% bupivacaine (60 mg) and 50 μg fentanyl were injected within 30 s.

In group S, after the patient was placed in the lateral decubitus position, spinal anesthesia was performed using 25 G spinal needle at the L3–4 interspace via midline approach. After cerebrospinal fluid appeared in the needle, 3 mL 0.5% hyperbaric bupivacaine (15 mg) and 50 μg fentanyl were injected intrathecally. After injection, the patient was placed in the supine position.

In group SA, after the patient was placed in the sitting position and free flow of cerebral fluid was observed, 3 mL 0.5% bupivacaine and 50 μg fentanyl were administered using 25 G spinal needle at the L3 - 4 via midline approach. After injection, the patient was remained in the sitting position for 5 mins.

### 2.3 Outcome measures

During the operation, 5 L/min oxygen was supplied *via* simple mask. Systolic blood pressure (SBP), heart rate, respiratory rate, and peripheral oxygen saturation were recorded at 5-min intervals. Intraoperative hypotension was defined as a 20% decrease from baseline or systolic blood pressure < 90 mg. When hypotension was observed, 5 mg of ephedrine was administered intravenously. Furthermore, 0.25 mg of atropine was administered intravenously when heart rate was < 50 beats/min.

Sensory block was assessed using pinprick test bilaterally on the midclavicular line every 5 min. Time to block of thoracic 10 (T – 10) dermatome, time to maximum sensory block, and 2-segment regression time were recorded. If the patient complained of pain during surgery, 50 μg of fentanyl was administered intravenously, and total amount of additional analgesics was recorded. General anesthesia was used if the patient had repeated complaint of pain.

In the recovery room after surgery, the motor block level was assessed using the modified Bromage scale (0 = no motor block, the patient can partially bend knees in supine position; 1 = partial motor blockade, the patient can move knees; 2 = almost complete motor blockade, the patient can move feet; and 3 = complete blockade, inability to flex ankle joints).

### 2.4 Sample size estimation

Based on the patients scheduled for HoLEP surgery, the papers conducted on TURP patients were used as the basis for the 3 group significance test. The primary outcome was the tie to block of the T – 10 dermatome. When the sample size was estimated and calculated based on the mean difference of the 9 minutes and standard deviation 15, 19 patients were considered necessary for each group. Estimating a 10% dropout rate, 20 patients were recruited for each group.

### 2.5 Statistical analysis

All data were analyzed using SPSS version 18.0. Demographic data, duration of operation, time to block of T - 10 dermatome, time to maximal sensory block, and 2-segment regression time were assessed using one-way analysis of variance. Chi-square test was used to compare the Bromage scores. A *P* value < .05 was considered statistically significant.

## 3 Results

The demographic characteristics of age, weight, and American Society of Anesthesiologists Physical Status score were similar for all groups. Furthermore, differences were not observed between the groups in terms of duration of surgery, preoperative and postoperative sodium level, and total volume of irrigating fluid (Table 1). In addition, statistical difference was not observed in preoperative and postoperative sodium levels in each group.

**Table 1:** Patient characteristics and operation details.

	Group E (n = 20)	Group S (n = 20)	Group SA (n = 20)	*P* - value
Age (yr)	70.1 ± 5.6	70.4 ± 6.3	74.0 ± 6.0	.08
Weight (kg)	66.6 ± 7.8	67.3 ± 9.1	61.4 ± 10.1	.09
Height (cm)	166.8 ± 4.9	163.5 ± 4.6	166.1 ± 5.7	.37
ASA I/II/III (n)	8/11/1	6/14/0	3/15/2	.30
Duration of surgery (min)	90.7 ± 39.5	82.9 ± 26.4	98.6 ± 44.6	.42
Pre- / postoperativeNa+ level (L/mEq)	140.7 ± 3.5 / 141.2 ± 1.9	141.7 ± 1.9 / 141.6 ± 1.9	140.9 ± 2.1 / 141.1 ± 2.3	.36/.75

A statistically significant difference was observed between groups E and S for time to reach T10 dermatome block (*P* = .013) and to maximal sensory level block (*P* = .006). Maximal sensory block times in group E were significantly longer than in groups S and SA (*P* = .023). In addition, maximal sensory block level was similar in group S and SA but higher in group E than in group SA (Table 2). There was a statistically significant difference in time to 2-segment sensory regression and was significantly longer in group E than in group SA (*P* = .006).

**Table 2:** Sensory block using regional anesthesia.

	Group E (n = 20)	Group S (n = 20)	Group SA (n = 20)	*P* - value
Time to T10 block (min)	10.4 ± 8.5^∗^	4.5 ± 3.8^∗^	7.2 ± 5.1	.01
Time to maximal level block (min)	26.1 ± 15.9^∗^^,^^†^	14.5 ± 7.7^∗^	16.3 ± 6.2^†^	.003
Maximal sensory block level	4.9 ± 1.5^†^	5.4 ± 1.7	6.7 ± 2.0^†^	.007
Time to two-segment regression (min)	116.8 ± 26.5^†^	98.6 ± 22.3	93.8 ± 19.7^†^	.006

**Figure 1:**
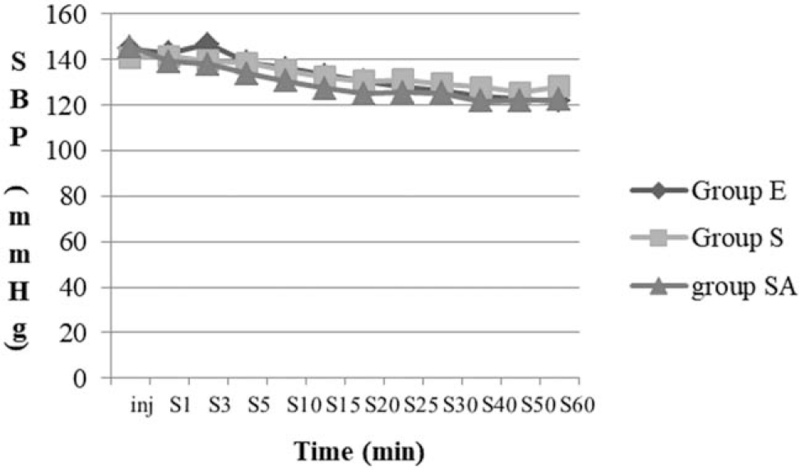
Changes in noninvasive systolic blood pressure over time. SBP = systolic blood pressure; Group E = epidural block; Group S = spinal anesthesia; Group SA = saddle block; Inj = time of drug injection; S = surgery.

**Figure 2:**
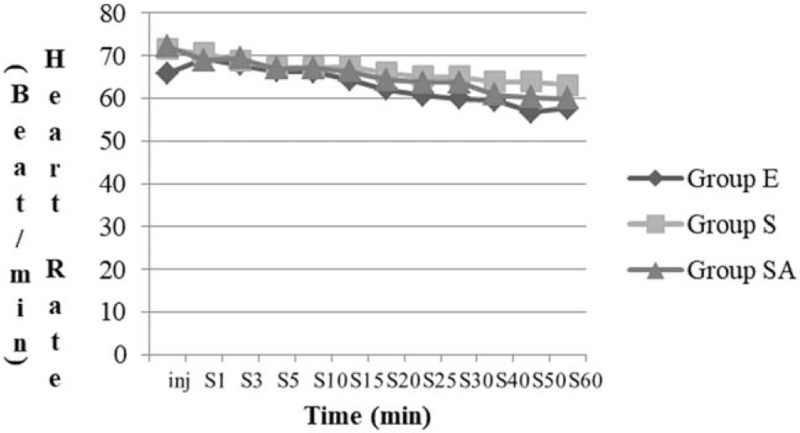
Changes in heart rate over time. Group E = epidural block; Group S = spinal anesthesia; Group SA = saddle block; Inj = time of drug injection; S = surgery.

Table 3 shows the distribution of Bromage scores. A statistically significant difference was observed in terms of motor block values (*P* < .05); however, significant difference was not observed in systolic blood pressure and heart rates among the 3 groups (Figs. 1 and 2).

**Table 3:** BS distribution among groups.

	Group E (n = 20)	Group S (n = 20)	Group SA (n = 20)
BS 0,1	13 (65)	2 (10)	8 (40)
BS 2,3	7 (35)	18 (90)	12 (60)

## 4 Discussion

HoLEP has become an appealing technique with the same indications as TURP. The accumulated results show low morbidity and effective clinical results.^[11,12]^ In the present study, regional anesthesia procedures for HoLEP were compared.

Pain signal from bladder distension travels from T11 to L2 sympathetic fibers. Considering this innervation, height of regional block up to T10 is sufficient for TURP operation. In the present study, the height of the block in all 3 techniques crossed the T10 level and provided sufficient anesthesia for HoLEP.

Tuominen^[13]^ reported the major factors affecting the distribution of local anesthetics to be concentration and volume of local agent and position of the patient during and after the injection. It is important to use a stable anesthesia method with minimal hemodynamic changes. Hypotension occurs in approximately 30% of patients during spinal anesthesia^[14]^ and it results from sympathetic tone block and large intravascular volume shifts due to venous dilatation. In the present study, most patients in the 3 groups showed stable hemodynamic status, and significant difference was not observed in terms of heart rate and systolic blood pressure. In several studies, use of low-dose local anesthetics with low dose of fentanyl in spinal anesthesia was reported to reduce the risk of hypotension.^[15,16]^ Fentanyl is a lipophilic opioid agent with small molecular weight and high potency. Intrathecal fentanyl as an adjuvant can enhance sensory blockade without changing the degree of sympathetic blockade.^[17]^

Furthermore, the minimal amount of time the patient remained in sitting position (5 min) in group SA might have also affected the sensory block and systolic blood pressure results between groups S and SA. Bupivacaine provides sufficient anesthesia at 15 mg dosage, and maintaining a sitting position for 2 to 3 minute after the injection might be sufficient for anesthetic distribution.

Controlled hypotension is a technique used to limit intraoperative blood loss to provide the best possible field for surgery.^[18]^ It can be indicated in HoLEP, and the benefits include reduction in blood loss with improved quality of surgical field. As mentioned above, a decrease in SBP after neuraxial block is common, and it can be an advantage in the controlled hypotension technique. In this study, SBP decreased by an average of 34.73 ± 16.59 mm Hg after local anesthetics injection. Although there were no differences in maximal fall of SBP among the 3 groups, decrease of SBP were statistically significant in all 3 groups (*P* < .05).

The advantage of HoLEP is from the decreased amount of irrigation solution required. Furthermore, whether the irrigant can conduct electricity is irrelevant, and normal saline can be used. TURP syndrome is from water intoxication, and occurs when hypotonic irrigating fluid is absorbed to produce systemic manifestations. The incidence of mild to moderate TURP syndrome is reportedly between 0.5% to 8%.^[19]^ Severe TURP syndrome is rare; however, the mortality rate can be as high as 25%.^[20]^ The early symptoms and signs are vague and nonspecific; thus, recognition and diagnosis in the early phase are important. The subarachnoid block is the most commonly used anesthetic technique in TURP, due to rapid detection of early symptoms such as change in mental status. Minimal fluid absorption eliminates the risk of classical TURP syndrome. In the present study, significant decrease was not observed between preoperative and postoperative sodium values in all 3 groups. A general anesthetic with short-acting inhaled anesthetic gases or even IV sedation is desirable because the systemic complications of TURP syndrome are diminished. Future, comparative studies between general anesthesia and regional anesthesia for HoLEP are needed.

HoLEP surgery is technically demanding and requires a long and steep learning curve of approximately 50 cases, which can be reduced to 27 under expert supervision.^[11]^ Procedure times for enucleation of the prostate are longer for the HoLEP compared with classic TURP.^[21,22]^ In the present study, the average operation time was more than 90 minute, and the 2-segment sensory regression time was more than 90 minute in all 3 groups. HoLEP in the learning phase requires more time than if performed by an experienced surgeon. Soto-Mesa et al^[6]^ reported the rate of conversion from spinal anesthesia to general anesthesia in the learning phase to be higher than in the stabilization phase. Therefore, choosing the method with an appropriate length of anesthesia necessary for an extended surgical procedure time is still important. When the surgical team is in the learning phase, epidural block with long sensory regression time (116.8 ± 26.5 min) might be considered, but the possibility of switching to general anesthesia should also be considered. Therefore, it is important to predict the length of operation and to consult with an experienced anesthesiologist. The size of prostate can also be a factor in predicting the operation time along with the experience of the surgeon.

Adequate recovery and early mobilization after surgery are important factor for length of hospital stay. Intrathecal fentanyl as an adjunct to spinal anesthesia has been reported to increase the quality of spinal anesthesia and enhance anesthesia without extending sensory or motor recovery time or length of hospital stay.^[21]^ In the present study, use of the epidural block was associated with weaker motor block after surgery; however the 2 segment sensory regression time was significantly longer in group E. Conversely, in group SA, faster motor and sensory recovery were obtained compared with the other groups.

In conclusion, we suggest that saddle block induced by the combination of 15 mg 0.5% bupivacaine and 50 μg fentanyl is preferable due to rapid sensory regression time and weaker postoperative motor blockade with stable hemodynamic changes. More rapid onset, effective sensory block, and fester recovery were observed with saddle block.

## Author contributions

**Conceptualization:** Yumi Kim.

**Data curation:** Eun Hwa Jun.

**Formal analysis:** Eun Hwa Jun.

**Investigation:** So Young Kwon, Seung Tae Choi, Yumi Kim.

**Methodology:** Young Hoon Kim.

**Project administration:** So Young Kwon.

**Supervision:** So Young Kwon.

**Visualization:** Seung Tae Choi, Seong Jin Park.

**Writing – original draft:** Young Hoon Kim.

**Writing – review & editing:** Yumi Kim.
